# Optimizing the depth and the direction of prospective planning using information values

**DOI:** 10.1371/journal.pcbi.1006827

**Published:** 2019-03-12

**Authors:** Can Eren Sezener, Amir Dezfouli, Mehdi Keramati

**Affiliations:** 1 Bernstein Center for Computational Neuroscience Berlin, Berlin, Germany; 2 Technische Universitaet Berlin, Berlin, Germany; 3 Data61, CSIRO, Australia; 4 School of Psychology, UNSW, Sydney, Australia; 5 Gatsby Computational Neuroscience Unit, Sainsbury Wellcome Centre, University College London, London, UK; 6 Max Planck Centre for Computational Psychiatry and Ageing Research, University College London, London, UK; University of Pennsylvania School of Arts and Sciences, UNITED STATES

## Abstract

Evaluating the future consequences of actions is achievable by simulating a mental search tree into the future. Expanding deep trees, however, is computationally taxing. Therefore, machines and humans use a plan-until-habit scheme that simulates the environment up to a limited depth and then exploits habitual values as proxies for consequences that may arise in the future. Two outstanding questions in this scheme are “in which directions the search tree should be expanded?”, and “when should the expansion stop?”. Here we propose a principled solution to these questions based on a speed/accuracy tradeoff: deeper expansion in the appropriate directions leads to more accurate planning, but at the cost of slower decision-making. Our simulation results show how this algorithm expands the search tree effectively and efficiently in a grid-world environment. We further show that our algorithm can explain several behavioral patterns in animals and humans, namely the effect of time-pressure on the depth of planning, the effect of reward magnitudes on the direction of planning, and the gradual shift from goal-directed to habitual behavior over the course of training. The algorithm also provides several predictions testable in animal/human experiments.

## Author summary

When faced with several choices in complex environments like chess, thinking about all the potential consequences of each choice, infinitely deep into the future, is simply impossible due to time and cognitive limitations. An outstanding question is what is the best direction and depth of thinking about the future? Here we propose a mathematical algorithm that computes, along the course of planning, the benefit of thinking another step in a given direction into the future, and compares that with the cost of thinking in order to compute the net benefit. We show that this algorithm is consistent with several behavioral patterns observed in humans and animals, suggesting that they, too, make efficient use of their time and cognitive resources when deciding how deep to think.

## Introduction

“*There is proportional value in our attention to each action—so you will not lose heart if you devote no more time than they warrant to matters of less importance*.”

– Marcus Aurelius, *Meditations* [1]

When confronted with several choices, we need to have an evaluation of how good each option is. Each choice has some immediate consequences, but also takes us into a new state where new choices emerge, and so on. Think of chess as an example. One intuitive way to solve a sequential decision-making problem like chess is to prospectively think into the future. This idea, known as model-based planning in the reinforcement learning literature [2], expands a mental decision-tree by simulating a number of future action sequences. Although this method is accurate (in terms of statistical efficiency), evaluating deep trees is computationally expensive (in terms of time, working memory, metabolic energy, etc.). In chess, for example, it is impossible even for the best supercomputers to expand the tree of all possible strategies up to the end of the game. Therefore, several solutions have been provided in the artificial intelligence literature for how to approximate the values of choices without expanding a search tree to its fullest extent [3] or how to make the best use of limited computational resources to plan better [4].

To avoid the costs of planning altogether, a drastic alternative is to rely on heuristic methods that evaluate choices without any tree expansion. For example, a chess player can evaluate a chess position, without investigating the possibility of that position leading to a win or lose, by simply counting up the values of their pieces—a common heuristic utilized by novice players. Another example of approximate evaluation techniques, widely used in both natural and artificial intelligence. is using habits. This method, known as model-free reinforcement learning [2, 5], simply “caches” the average of previously realized rewards ensued by performing each action, and uses the cached values for evaluating those choices should they come up again in the future. Although using such heuristics frees cognitive resources from model-based planning, the downside is their inaccuracy. Habits, for example, take many trials to form, and they are always unreliable in changing environments.

Rather than clinging to one of these extreme solutions (i.e., full planning vs. heuristics/habits), an intelligent agent can instead combine the two in order to harvest the relative advantages (i.e., accuracy vs. affordability) of both techniques [6–9]. This, in theory, is achievable by forward planning up to some depth and then exploiting heuristic values as proxies for consequences that may arise in the further future. That is, when the depth of planning is say *d*, the agent computes the value of a choice by adding the first *d* rewards predicted by explicit simulation, to the value of the remaining actions estimated by the heuristic/habitual values. For example, a chess player could think three steps ahead, and then estimate, heuristically, the strength of the position he could achieve after those three moves. This integrative approach has been used in artificial intelligence for example for obtaining super-human Go performance [10]). Furthermore, it was shown recently that humans also use this scheme, named plan-until-habit, for integrating planning and habitual processes in a normative way, and that their depth of planning depends on the time-pressure imposed on them [11].

The plan-until-habit (or plan-until-heuristic, in general) scheme aims at mitigating the computational costs of planning by appealing to the habitual system after the planning system has *sufficiently* expanded the decision-tree. Obviously, the first questions to be asked in this framework are “in which directions the decision-tree should be expanded?”, and “when should the expansion stop?”. In this paper, we present, for the first time, a principled algorithm for optimal tree-expansion in the plan-until-habit framework. The algorithm is based on a speed/accuracy tradeoff: deeper planning leads to more accurate evaluations, but at the cost of slower decision-making. As a proof of concept, we show through simulations how this algorithm expands the decision-tree effectively and efficiently in a simulated grid-world environment. We further show that our algorithm can explain several behavioral patterns in animals and humans, namely the effect of time-pressure on the depth of planning, the effect of reward magnitudes on the direction of planning, and the gradual shift from goal-directed to habitual behavior during training. The algorithms also provide several predictions testable in animal/human experiments.

## Results

### Theory sketch

From an *external-observer* viewpoint, the questions to be answered by an agent are of the type “what action should be taken?”. From a *metacognitive* perspective, however, the agent should first think about how to think (e.g., how deep she should plan). In fact, the question she could ask at each step of the planning process is “Should I expand the decision-tree one step further?”, and if yes, “In what direction?”.

**Fig 1:**
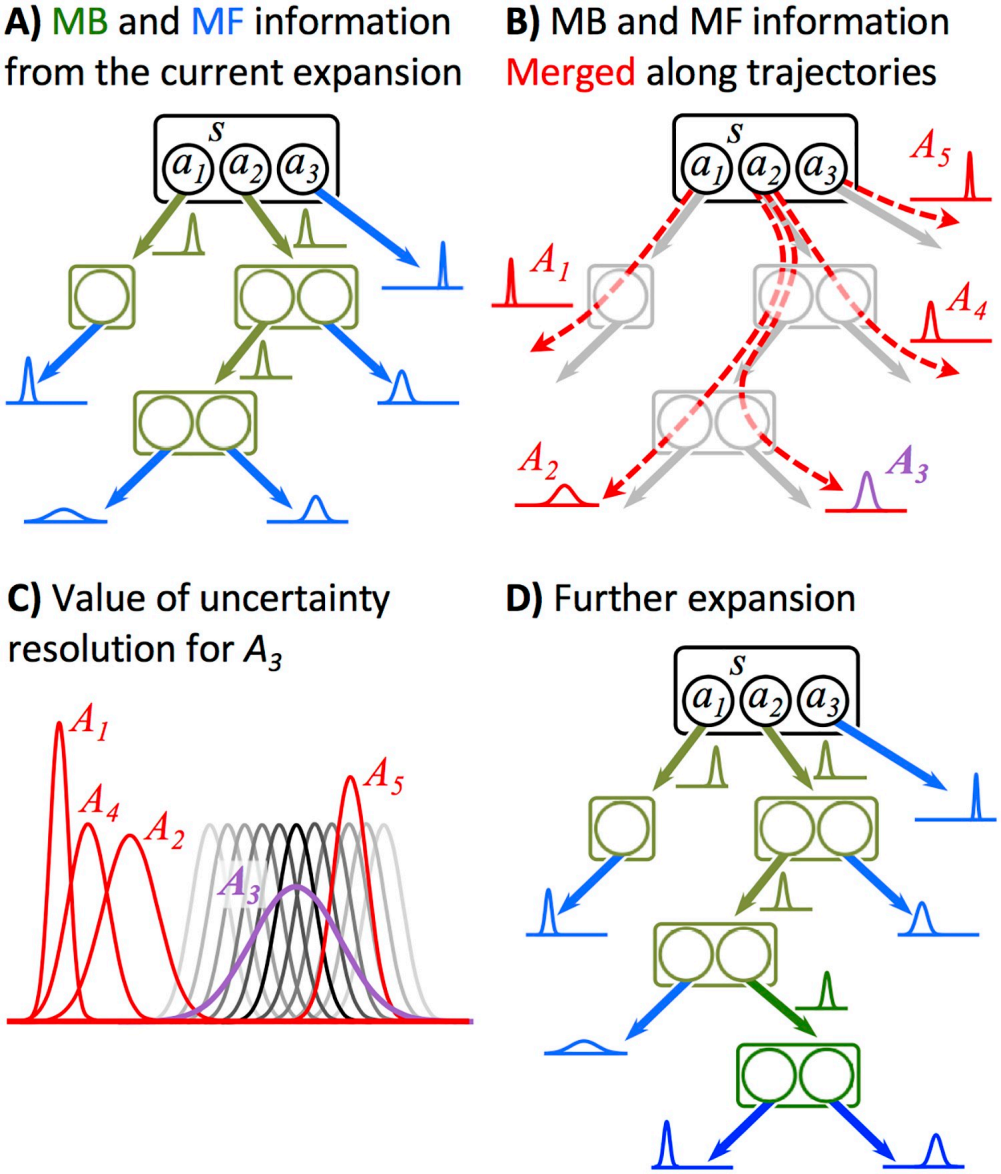
Overview of the pruning scheme, illustrated via an example. (A) A snapshot of the search tree. Nodes of the tree represent states, and each state has a number of available actions, denoted with circles, that lead to next states. Blue graphs show value distributions for the leaves of the tree, estimated by the model-free (MF) or any other heuristic system. Green graphs show the immediate rewards for previously expanded state-actions, estimated via the model-based (MB) system. (B) Each path from the root to a leave forms a strategy, *A*_*i*_, with a corresponding value distribution. These distributions are obtained by summing up the value distributions of the leaves with the immediate reward distributions accumulated along the way. (C) To compute the value of uncertainty resolution (vur), say for *A*_3_, the agents assumes that one further expansion would result in a sharper value distribution (one of the black/grey distributions). The location (i.e., the mean) of the new distribution cannot be known in advance, but it can be treated as a random variable, whose distribution can be analytically obtained (Eq 14). The vur for *A*_3_ is therefore the expected value, over all possible sharper distributions (grey curves), of the additional rewards that can be obtained by a policy improvement in the light of that potential new information (i.e., the sharper distribution). (D) After computing vur for all strategies *A*_*i*_, the highest vur (in this case, for *A*_3_) is compared to the cost of expansion. If it is bigger than the cost, the tree expands along the direction of that strategy. This corresponds to loading a new node, which is the successor state of the leaf of *A*_3_, from the MB system and adding it to the tree.

To answer these, assume that the agent has already expanded a tree to a certain extent (Fig 1A). This means that the agent knows, possibly with some uncertainties, a few next states to be visited upon taking each action, and the immediate rewards associated with each of those transitions. She can, therefore, sum up the predicted rewards along each trajectory (i.e., action-sequence) and have an estimate of the total rewards to be achieved. On the top of this “total immediate rewards”, each trajectory ends in a frontier state which represents the edge of the current planning horizon along that trajectory. The habitual (or any other heuristic) values on this frontier state supposedly reflect the total (discounted) rewards to be expected from that point on. Therefore, the sum of “total immediate rewards” and the habitual value of the frontier node provides an estimate of the total expected reward of each trajectory (Fig 1B).

Habitual values, however, can be highly unreliable due to the inflexible nature of habit formation. For each given trajectory, therefore, the dependence of its estimated total rewards on uncertain habitual values renders the whole estimation uncertain. If expanding the tree along that trajectory would make value estimation less dependent on habitual values and thus reduce uncertainty, that expansion is worth considering. In this sense, the critical value to be computed for each trajectory is the “value of uncertainty reduction” (vur). vur computation for a trajectory should examine whether a new piece of information, possibly providable by a further expansion of the tree along that trajectory, could change agent’s decision about what action to be taken, and how much extra value is expected to be gained by that policy improvement. vur is, in fact, the expected value of policy improvement-induced rewards, computed over all possible new pieces of information that could be provided by expanding the trajectory one step further (Fig 1C). Although the agent readily possesses those new pieces of information in her memory (because she has a model of the environment), loading them into working memory and taking them into the value-estimation account is worth doing only if the value of uncertainty reduction is more than its cost.

Here is the general scheme of our algorithm: at each stage of planning, vur is computed for each trajectory on the search tree (we discuss later that previously-computed vur-values can be reused later under certain conditions). The trajectory with the highest vur is expanded if its vur is bigger than the cost of expansion. Otherwise, the expansion process is terminated and the agent chooses an action (e.g., using soft-max rule) according to the estimated values derived from the tree.

In this paper, we assume that the cost of expansion simply reflects the opportunity cost of time. That is, assuming that each expansion takes *ϵ* time units, the total cost of one expansion is $\bar{R}\epsilon$, where $\bar{R}$ is the average reward the agent receives in the given environment.

As explained before, the main motivation for expanding the tree is reducing value-estimation uncertainties. There could be several reasons for why expansion reduces uncertainty. In many cases, like chess, heuristic estimations become more precise as the game advances. In general, proximity to goal sometimes makes it easier to evaluate the states. Another way that expansion reduces uncertainty, which is the focus of our formal model, is through temporal discounting. By each level of expanding a trajectory, the dependence of its estimated value on the less-reliable habitual system is shifted one step further into the future.

As a simplified example, imagine you are in a maze and you have already thought two steps ahead along a certain trajectory, *T*_1_, of actions, and those two steps will take you to the state *s*′. You can use the MF value, *V*_MF_(*s*′) of that state to compute the total value of the trajectory: *V*(*T*_1_) = *r*_1_ + *γ*.*r*_2_ + *γ*^2^.*V*_MF_(*s*′), where *r*_1_ and *r*_2_ are the immediate rewards expected to be received by performing the first and the second actions on the trajectory *T*_1_. Assuming that the estimates of the immediate rewards have zero uncertainty, and that the MF estimates always have variance *σ*^2^ (i.e., uncertainty)), the total uncertainty of *V*(*T*_1_) will be (*γ*^2^.*σ*)^2^ = *γ*^4^.*σ*^2^. Now, if you think one step deeper and expect to land in state *s*^′′^ after taking the first three steps of trajectory *T*_2_, then *V*(*T*_2_) = *r*_1_ + *γ*.*r*_2_ + *γ*^2^.*r*_3_ + *γ*^3^.*V*_MF_(*s*^′′^). Therefore, its variance will be (*γ*^3^.*σ*)^2^ = *γ*^6^.*σ*^2^. This toy example shows that as a natural consequence of temporal discounting, by increasing the depth of planning, the total uncertainty of trajectories decreases, due to the reduced reliance on uncertain MF values. Therefore, the discount factor is the critical variable that determines the extent of uncertainty reduction by each expansion.

In this paper, we only consider environments where the transition between states via actions are deterministic (i.e., deterministic transition function for the Markov decision process; See Methods for how this assumption can be relaxed). Therefore, the expanded tree, at each point, is a deterministic tree. In order to compute vur, let’s define a *strategy* in a tree as a combination of actions that an agent can take to reach a leaf in the tree (see Fig 1), and define a *frontier search* as the set of all strategies that agent can take in a given tree (e.g., the search frontier in Fig 1 is {*A*_1_, *A*_2_, *A*_3_, *A*_4_, *A*_5_}). Based on this definitions, as shows in the Methods section, the value of uncertainty reduction for strategy *A*_*i*_, given the search frontier *F*, can be written as:

$$\begin{array}{c} \text{VUR}\left(A_{i}|F\right)=\underset{\text{with}\;\text{expansion}}{\underset{︸}{E_{\mu_{i}^{*}}[\max(\mu_{i}^{*},\max_{A\in F-A_{i}}E[V(A)])]}}-\underset{\text{without}\;\text{expansion}}{\underset{︸}{\max_{A\in F}E[V(A)]}}\;, \end{array}$$ (1)

where *F* − *A*_*i*_ is the set *F* excluding *A*_*i*_. According to this equation, computing vur(*A_i_*|*F*) requires $\mu_{i}^{*}$, which is the expected mean of strategy *A*_*i*_ *after* the potential expansion. However, this variable can be computed before expansion, by $\mu_{i}^{*}∼N\left(\mu_{i},\left(1-\gamma^{2}\right)\sigma_{i}^{2}\right)$ (see Methods section), in which *γ* is the discount factor, and *μ*_*i*_ and $\sigma_{i}^{2}$ are respectively the mean and the variance of the MF-value distribution for the last action on *A*_*i*_. In other words, vur is computable based on $\mu_{i}^{*}$, the expectation with respect to the predicted value of *A*_*i*_ after expansion, instead of its realized value which is not available before the expansion (a more general form of the above equation without reliance on the discount factor is presented in the Methods section).

The right-hand side of Eq 1 is composed of two parts: the amount of future rewards that are expected to be gained with the expansion of strategy *A*_*i*_, and the amount expected to be gained without the expansion of *A*_*i*_. vur is the difference between these two quantities. The without-expansion term is simply the value of the best strategy that is currently available to the agent. In the with-expansion term, the outer ‘max’ operator implies that if after expanding, *A*_*i*_ turns out to be worse than the other available strategies (*F* − *A*_*i*_), then the best strategy among the other ones will be taken. Otherwise, *A*_*i*_ will be taken.

The agent, however, needs to calculate this term before the expansion of *A*_*i*_ and therefore the term is calculated based on the expectation with respect to the predicted value of *A*_*i*_ after expansion (denoted by $\mu_{i}^{*}$) instead of its realized value which is not available before the expansion.

It can be shown that in the case of normally distributed MF value functions, Eq 1 has a closed-form solution (see S1 Text for details):

$$\begin{array}{c} \text{VUR}\left(A_{i}|F\right)=\{\begin{array}{cc} \sigma_{i}\left[\phi \left(\frac{\mu_{i}-\mu_{\beta }}{\sigma_{i}}\right)-\frac{\mu_{i}-\mu_{\beta }}{\sigma_{i}}\Phi \left(-\frac{\mu_{i}-\mu_{\beta }}{\sigma_{i}}\right)\right]+\mu_{\beta }-\mu_{\alpha } & \text{if}A_{i}\;\text{is}\;\text{the}\;\text{best}\;\text{strategy}\\ \sigma_{i}\left[\phi \left(\frac{\mu_{i}-\mu_{\alpha }}{\sigma_{i}}\right)-\frac{\mu_{i}-\mu_{\alpha }}{\sigma_{i}}\Phi \left(-\frac{\mu_{i}-\mu_{\alpha }}{\sigma_{i}}\right)\right] & \text{otherwise} \end{array}\; \end{array}$$ (2)

where *μ*_*i*_ and *σ*_*i*_ are, respectively, the mean and the standard deviation of strategy *A*_*i*_. Furthermore, *μ*_*α*_ and *μ*_*β*_ are the means of the, respectively, first-best and second-best strategies in the currently-expanded tree. First-best and second-best strategies are the strategies that have the highest and the second-highest mean values. Finally, *ϕ* and *Φ* are, respectively, the probability density and cumulative distribution functions of a standard normal distribution.

A central principle for any meta-control algorithm is that the cost of meta-reasoning (here, the cost of computing arg max_*A*_ *VUR*(*A*|*F*)) should be lower than the cost of expensive reasoning (here, one-step expansion of the decision-tree). In terms of memory cost, tree-expansion would require loading information about the expanding nodes from the long-term to the working memory. Furthermore, it would require engaging an additional working memory slot to store such information. Meta-reasoning, however, has minimal memory cost, since all the variables for computing arg max_*A*_ *VUR*(*A*|*F*) already exist in the working memory (i.e., are in the already-expanded tree).

In terms of computational-time cost, we should stress that even though we want to find the strategy with the maximum vur value, this does not necessarily require computing vur’s of all strategies at each time step. vur(*A_i_*|*F*) only depends on *μ*_*i*_, *σ*_*i*_ and *μ*_*α*_ (or *μ*_*β*_). Therefore, vur values can be cached, and reused as long as the aforementioned parameters have not changed (i.e., the newly-added strategies are not first- nor second-best strategies). From an algorithmic point of view, computing vur of a given *A*_*i*_ can be viewed as a constant time operation. Therefore computing arg max_*A*_ vur(*A*|*F*) is in the order of O(|F|) in the worst case, where |*F*| is the cardinality of *F* (i.e., number of items in the search frontier). However, as shown in the appendix, as the tree expands, the expected cost becomes constant (i.e., O(1)) asymptotically, given that the agent caches previously computed vur values. This is intuitively becuase as the depth of the tree grows, the uncertainty around the value of the to-be-expanded strategy shrinks (becuase of the discounting factor), which makes it less likely that the strategy (which is not currently the best strategy) becomes the best one after expnasion (or second best strategy). As such, the chances that a new expansion affects previusly computed vur values becomes smaller and smaller as the tree gets deeper. This rate of decrement is faster than the rate at which new potential strategies are added to the tree as it gets deeper, and therefore overall the number of vur values that need re-computation remains constant as in the limit.

### Pruning in a grid world environment

Just as a proof of concept, we would like to see whether our method can be beneficial in a setting in which an agent is combining both MF and MB information for efficient planning. For this, we first trained an agent in an episodic grid-world environment where she obtains *imperfect* estimates of state-values by the model-free system. After training, she utilizes both the MF and the MB systems to use the plan-until-habit scheme, where the MB system is used to construct the tree, and the MF systems is used for estimating the values of state-actions that lie on the frontier of the tree. We predict that the increased accuracy in model-free estimates, as a result of training, would bias the direction of expanding the tree towards better states.

The agent starts each episode in the center of a 7 × 7 grid and can choose to go up, down, left, or right at each state. All the transitions are deterministic and are associated with a unit cost. The bottom right cell is the goal state that concludes the episode. This state is not associated with any reward, but is implicitly rewarding since it terminates the costly walk in the grid world. Evidently, the optimal policies are combinations of three right moves and three down moves. Given the structure of the task, for easier geometric interpretation and without loss of generality, the MF system learns state values, rather than state-action values.

To apply our plan-until-habit pruning algorithm, we require an MF system that learns not just the mean, but also the variance (i.e., uncertainty) over the state values. In our implementation, the agent estimates the value of a state by generating a number of trajectory samples from the state, similar to the first-visit Monte Carlo method described in [2], and utilizing the trajectories’ return statistics. However, instead of estimating the *Q*-values with Monte Carlo averages, we use independent conjugate normal priors and obtain posterior estimates of *Q*’s, which are conditioned on the trajectory returns (see S1 Text). We obtain *N* trajectory samples starting from each state, such that each sample consists of a trajectory resulting from a fixed uniform random policy that assigns $\frac{1}{4}$ probability to each direction {UP, DOWN, LEFT, RIGHT}.

We test our planning model in two different settings. First, we assume the agent has no experience interacting with the environment (i.e., *N* = 0). This condition results in the posterior *Q*-values having large and equal variances. We compare this with the case where the agent has collected some samples (i.e., *N* = 10), resulting in more accurate estimates of state values. In both cases, we employ the same pruning mechanism, with a variable number of possible tree expansions (capturing working-memory limitations; see Discussion section) selected uniformly from [5, 25] and *γ* = 0.95.

**Fig 2:**
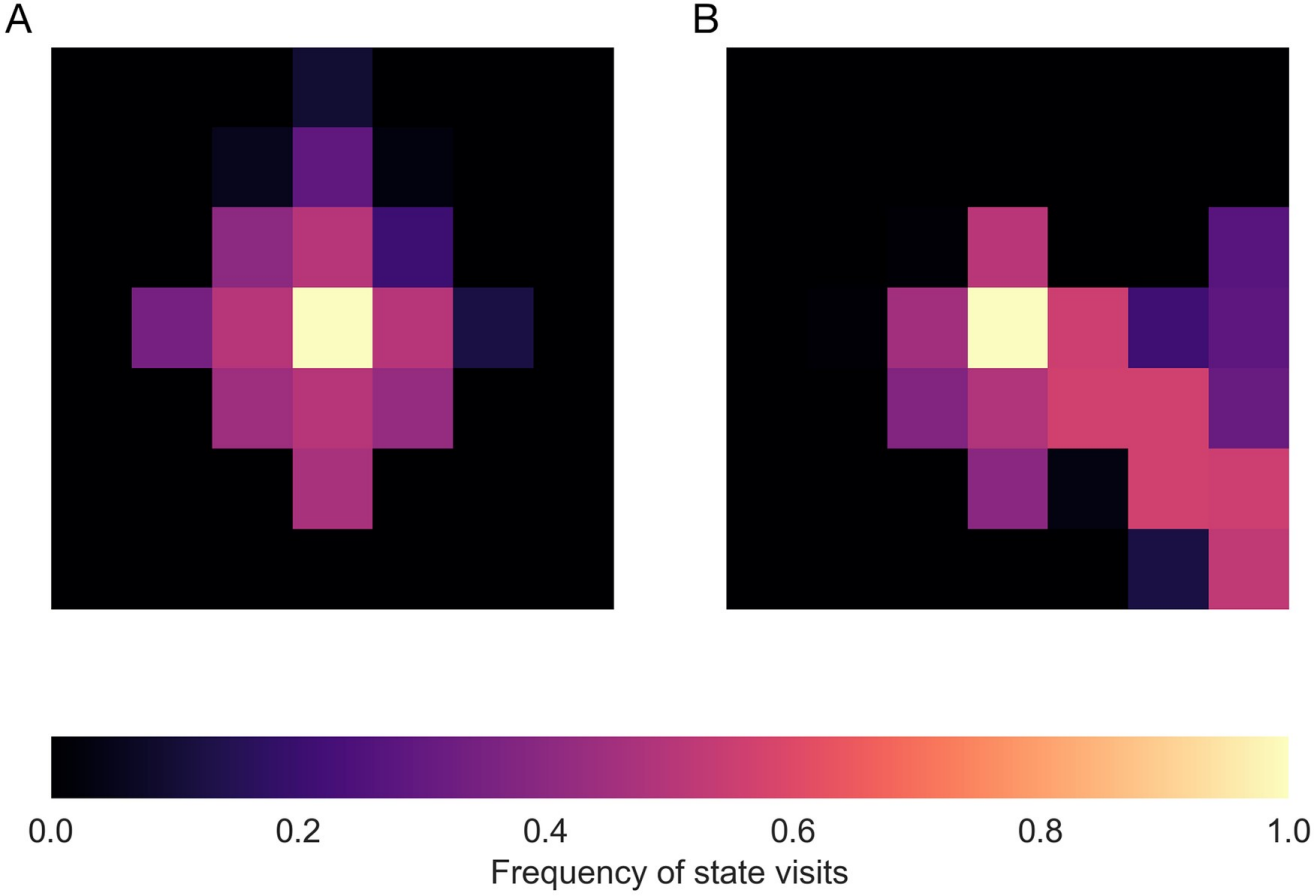
Grid-world pruning simulation results. Reaching the bottom-right corner of the map with minimum moves is rewarding. The heatmaps show the frequencies of state-visits during the tree expansion when the agent starts from the middle of the map, and (A) the agent has had no prior exposure to the environment, or (B) after some exposure (i.e., 10 trajectory samples from each state) resulting in more accurate estimates of model-free values.

As displayed in Fig 2A, in the no-experience condition, the search tree is explored in all directions almost uniformly. In the second condition, however, the search is directed more towards the goal state as illustrated in Fig 2B. These results are in line with our intuition that the agent prunes more aggressively as she gathers more experience and thus, is better able to judge what the promising states or actions are.

### Human-like pruning

Behavioral evidence suggests that humans, when planning, curtail any further evaluation of a sequence of actions as soon as they encounter a large punishment on the sequence [12]. In a behavioral task [12], subjects were required to plan ahead in order to maximize their income gain. The environment in the task is composed of six states. Each state affords two actions, each of which transitions the subject to another state deterministically. Subjects see their current state on a display and press the ‘U’ or ‘I’ buttons on the keyboard to transition to a different state.

**Fig 3:**
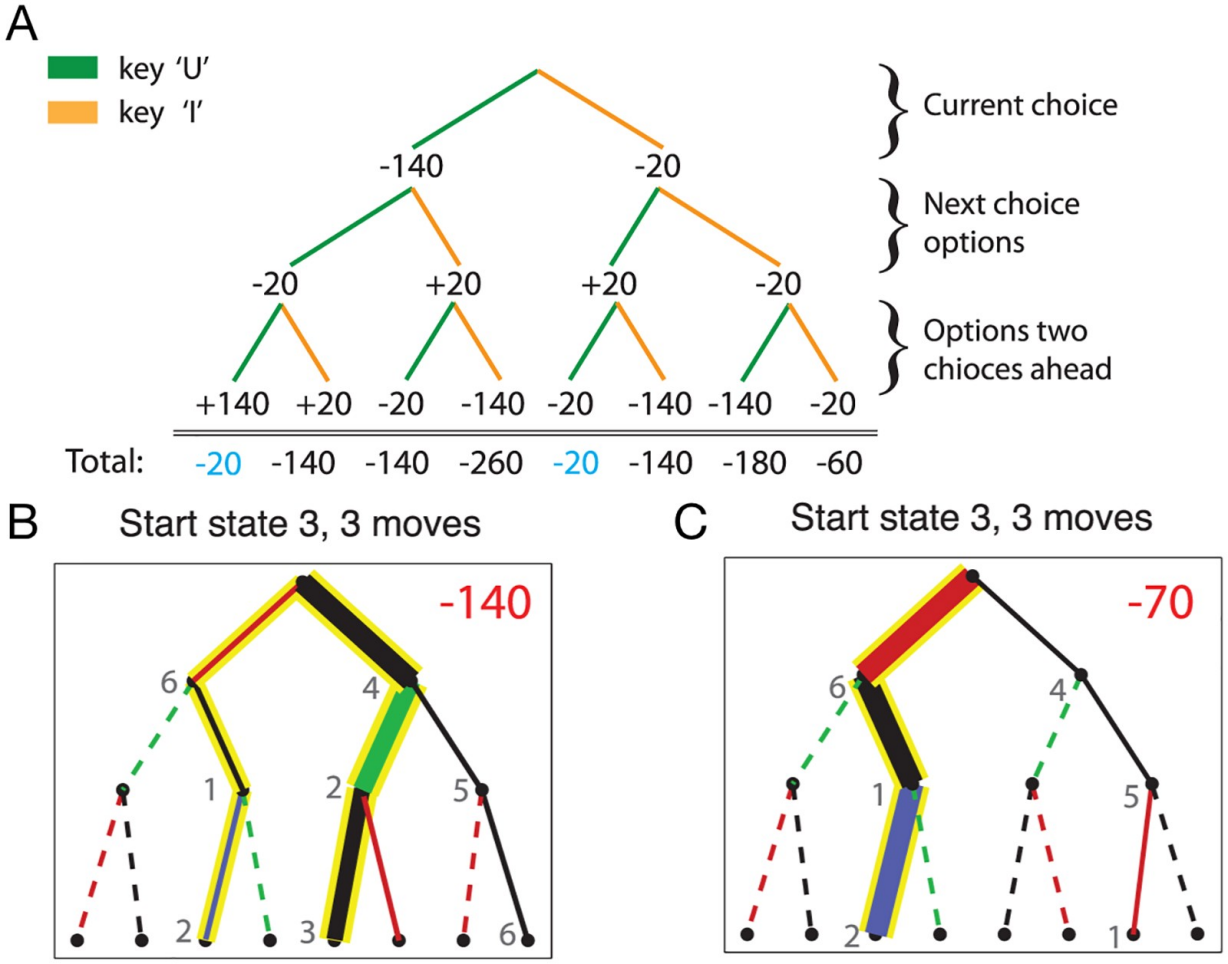
Example search trees from [12]. **A**: Starting at state 3, subjects make three consecutive decisions (pressing ‘U’ or ‘I’), each of which are associated with a gain or loss. Two trajectories maximize the cumulative rewards in this example and achieve −20. **B** and **C**: State transition frequencies of subjects. Higher frequencies are illustrated with thicker lines. If a transition is not taken by any of the subjects, then it is illustrated with a dashed line. Yellow backgrounds show the optimal trajectories. Colors red, black, green, and blue denote the transition rewards of *P*, −20, + 20 and + 140 respectively. **B**: *P* = −140 condition. It can be seen that the subjects avoid the action associated with the large punishment. **C**: *P* = −70 condition. Subjects are eager to take transitions with large losses when such transitions lead to large gains (i.e., + 140), which in fact is the optimal strategy. Reprinted with permission from [12].

In the first phase of the experiment, subjects learn the deterministic transition structure of the environment. In the second phase, transitions are associated with specific gains or losses, which are visually cued to make it easier to remember. At each trial in this stage, subjects are told to take a certain number of actions, varying between 2 and 8, and collect all the rewards and punishments along their chosen trajectory. This forces them to think ahead and plan in order to find a relatively profitable trajectory among 2^2^ = 4 to 2^8^ = 256 options. For example, in the setting described in Fig 3A, 8 possible trajectories resulting from 3 consecutive actions are displayed.

Out of all 12 transitions, 3 of them are associated with a large loss. The magnitude of this loss is manipulated across trials (from {−140, −100, −70}) such that for certain losses (i.e., −100 and −70), Pavlovian pruning results in suboptimal strategies. In other words, pruning a strategy that starts with a −100 or −70 loss would result in discarding the most profitable course of actions, since such actions will eventually lead to highly rewarding states. The results of this experiment show that humans prune infrequently if pruning results in prematurely discarding optimal trajectories. Conversely, they tend to prune liberally when pruning does not eliminate the optimal trajectories. That is, they prune more when the loss on a trajectory is so large (i.e., −140) that cannot be compensated for by future rewards.

We aimed to replicate this task in our simulations. Because in the first part of the experiments subjects learn the transition and the immediate rewards through repetitive exposure, we assume that the agent (i.e., our simulation of a subject) knows the transition and reward structures. Since the immediate state-action rewards are visually cued, subjects, after observing their starting state *s* and their available actions *a*_1_ and *a*_2_, presumably incorporate the immediate rewards of those actions into their planning at no cost. Therefore, we assume that the agent starts the decision tree with two already-expanded actions, with values *Q*(*a*_*i*_) = *R*(*s*, *a*_*i*_) + *γV*(*T*(*s*, *a*_*i*_)), where *i* ∈ 1, 2, and *R*(*s*, *a*) and *T*(*s*, *a*) are the immediate reward and successor states resulting from taking action *a* at state *s*.

As in the previous experiment, we obtain the posterior *Q*-value distributions of the agent through a training stage. Similar to the training phase of the original study, we have the simulated agent interact with the environment for 100 episodes, during which she observes transitions and collects reinforcements. At each trial, the agent is located in a random state and is allowed to make a certain number of moves, which is sampled uniformly from {2, 3, 4}. She selects actions following uniform random policy, and stores the mean cumulative reinforcements collected after taking action *a* at state *s*, similar to the first-visit Monte Carlo algorithm [2]. Those mean values are then used for obtaining the posterior *Q*-distributions assuming a conjugate normal distribution as in the previous experiment (see S1 Text). The prior is a normal distribution with mean and standard deviation of 0 and 1000, respectively. After the training stage, the agent moves on to the pruning state, where she starts at state *s* and is asked to mentally expand the planning tree for *n* ∈ {2, 4, 6, 8, 10, 12*s*} steps. We record the frequency with which the agent expands the early branch with the large punishment, which we very between −40 and −140. Finally, we set *γ* to 0.95 as before.

**Fig 4:**
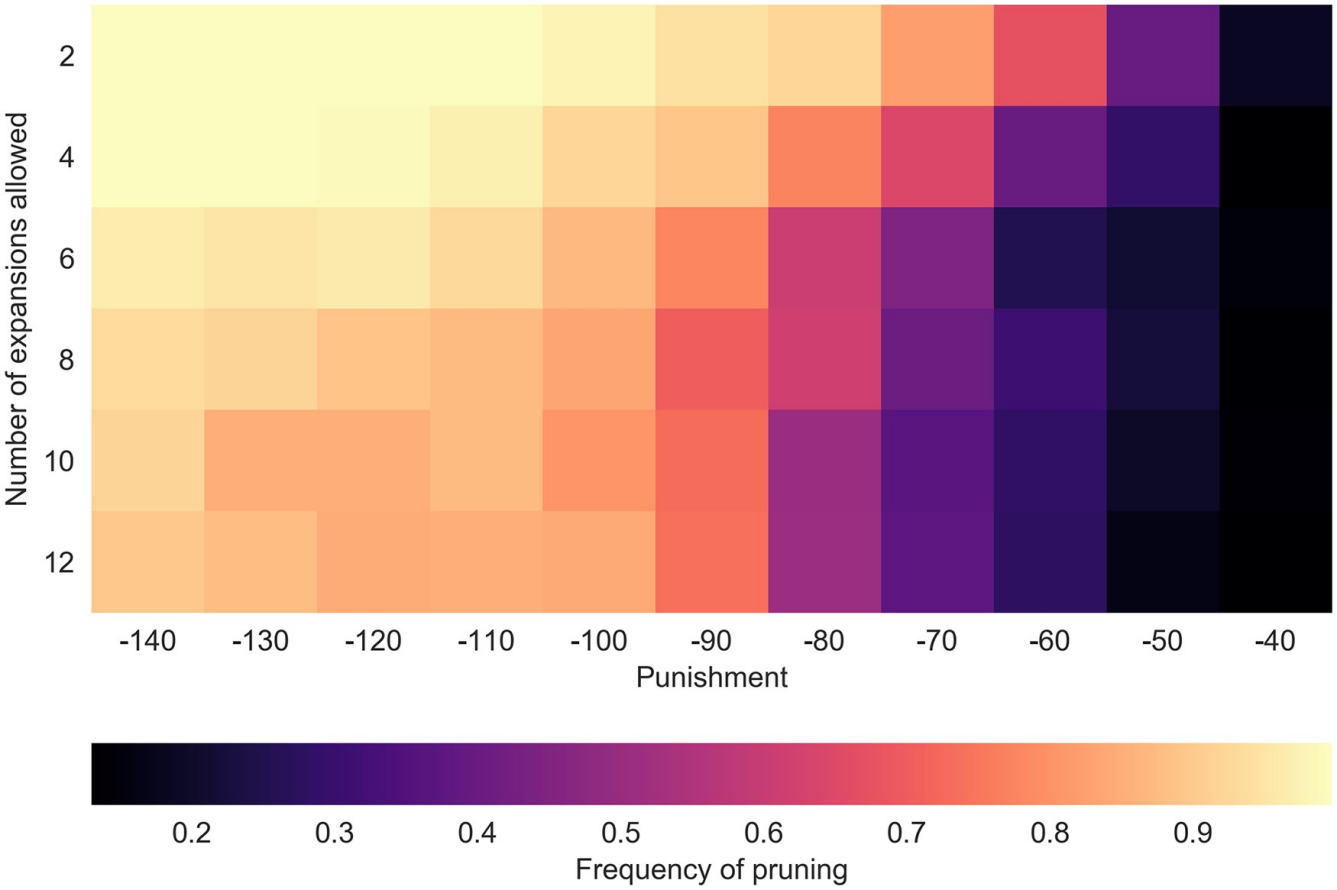
The frequency of pruning the branch with the large punishment. The black area on the right is the region where the agent does not prune (i.e., expands) the punishment branch. Each condition is averaged over 300 simulations.

One critical observation in [12] is that subjects prune more frequently as the magnitude of the punishment increases. As shown in Fig 4, our simulation results account for this pattern. Intuitively, observing a punishment on a trajectory reduces the expected value of the trajectory and thus, reduces the overlap between the value-distribution of that trajectory and that of the best trajectory. When the punishment is large enough, the overlap becomes very small even if the trajectories have highly uncertain value estimates. Small overlap is equivalent to low “value of uncertainty resolution” expected from expanding the unpromising trajectory, because there is a very small chance that the new pieces of information will render the unpromising trajectory better than the currently best strategy.

In the simulations, we also vary the maximum number of branches allowed to be expanded, reflecting constraints on the working memory capacity (see Discussion section). Not surprisingly, as the memory capacity is increased, pruning frequency decreases (Fig 4).

**Fig 5:**
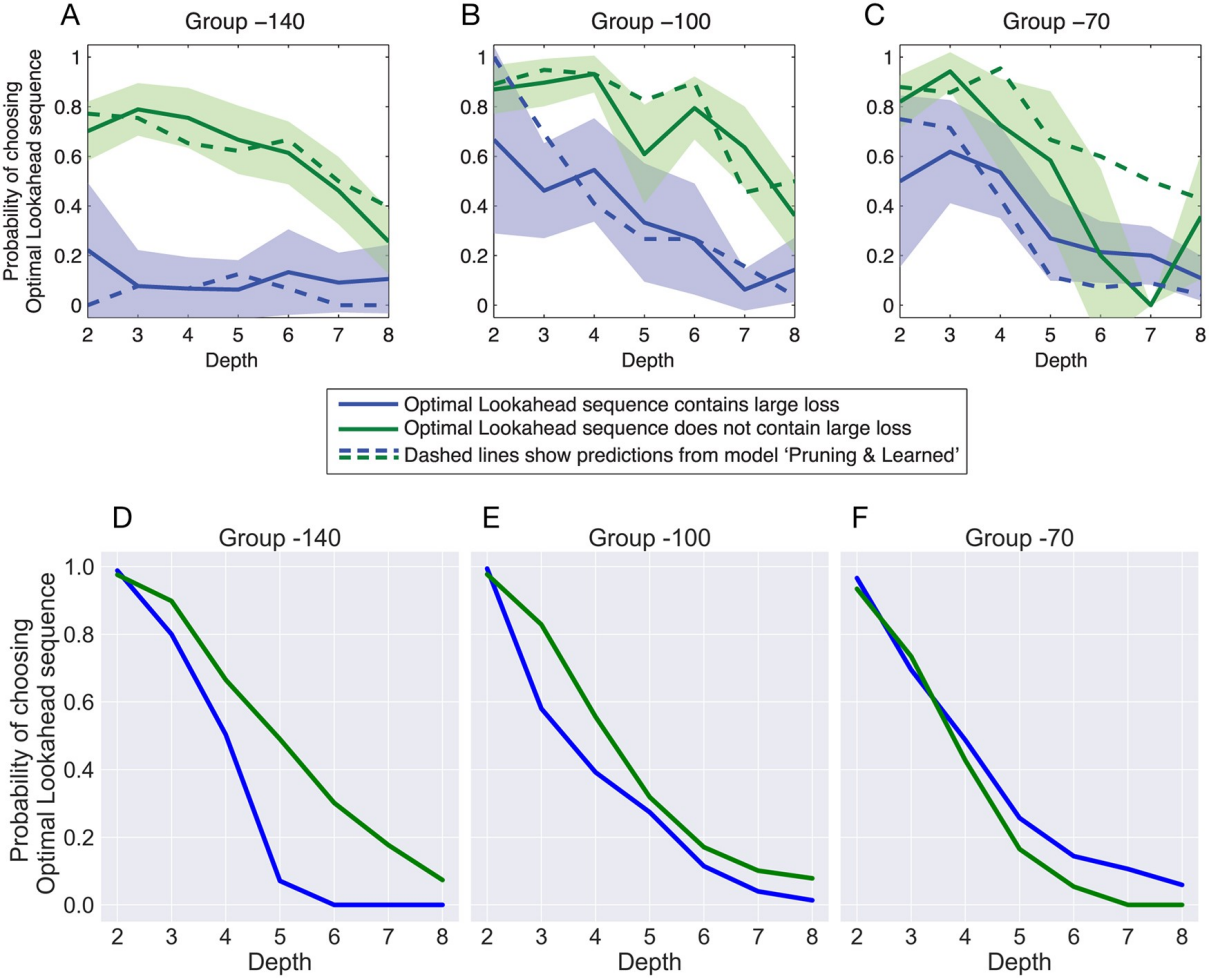
The top panels show the effect of different factors on choosing the optimal sequence of action. The panels are adapted from [12]. The x-axis denotes the number of actions the subjects were supposed to take, which determines the maximum depth of the search tree. The y-axis denotes the probability of choosing the Optimal Lookahead sequence. The blue lines represent the condition that the optimal sequences of actions included a big loss, and the green lines represent the condition that the optimal sequence of actions did not include a big loss. The amount of big loss is varied among the panels, and is mentioned by Group X on top of the panels, in which X denotes the amount of big loss (X = -140, -100, -70). The bottom panels are similar to the top panels but using the data obtained from the simulations of the model in the same settings.

Another important aspect of the study is that the likelihood of selecting the optimal sequence of actions by the subjects was affected by three factors: (i) subjects were less likely to choose the “Optimal Lookahead” sequence when it contained a large loss, (ii) this effect became larger as the size of the loss increased, and (iii) the optimal sequence was more likely to be chosen when the tree was shallow (i.e., when the subjects were supposed to choose a smaller number of actions). These three effects are shown in the top panel of Fig 5 for the data reported in Huys et. al. [12]. The bottom panel displays the prediction of our method based on the simulations in the same task. It can be seen that similar to the actual data, we predict that the subjects will be more successful in picking the optimal sequence when it does not contain a large loss, the tree is shallow and the loss is small (i.e., the effect is strongest in the −140 group and the weakest in the −70 group). One notable qualitative mismatch between the top and bottom panels is that, our model assigns a higher probability of choosing optimal sequences for smaller depths than what is shown for the actual data on the top panel. This is because, in our setting, the agent is very likely to make enough expansions to find the optimal sequence for a tree of depth 2, as there are only 2^2^ = 4 possible sequences—which can be spanned with a small number of expansions. The number of expansions are sampled from round(Gamma(4, 2)) + 1, where + 1 ensures positivity. Given this distribution, it is often the case that the agent performs enough expansions to find the optimal. However, if we look at the top left plot in Fig 5, we see that the probability of choosing the optimal sequence is low if it contains a large loss—even for depth of 2. This might suggest that the subjects do not fully use their “expansion budgets”, if performing expansions do not seem advantageous. The same could be done in our scheme by stopping expansions altogether if the maximum vur is below a threshold. However, we refrained from doing so, and instead used a random number of expansions for simplicity, and for limiting the flexibility of the model to prevent overfitting. Other than this, all other parameters are kept the same as the ones used for generating Fig 3.

Previously, the punishment-induced pruning discussed here was explained assuming that a Pavlovian system, reflexively evoked by large losses, curtails further evaluation of the corresponding sub-tree [12, 13]. In our computational framework, however, this pruning pattern emerges naturally, rather than devising new mechanisms, from a speed-accuracy tradeoff. Furthermore, the normative nature of our explanation depicts punishment-induced pruning as an adaptive mechanism in the face of cognitive limitations, rather than depicting it an a “maladaptive” Pavlovian response [12].

### The effects of training and decision-making times on depth of planning

Several lines of research have shown a transfer of control over behavior from goal-directed to habitual decision-making during the course of learning [14–17]. Previous accounts of interaction between MB and MF algorithms [18, 19] explained this behavior by showing that the MF value estimates become more and more accurate along the course of experiencing a task. As a result, they eventually become more accurate than MB estimates [18], or become accurate enough that the extra information that MB planning can provide is not worth the cost of planning [19]. Therefore, a binary transition from goal-directed to habitual responding occurs in behavior.

Our model also explains the transition, but also suggests that it is gradual, rather than binary. As MF estimates become more accurate, the variance in strategy values decrease and thus, vur values also decrease monotonically (see S1 Text for an analytical proof of this effect). This implies that an experienced agent would construct a shallower search tree and hence, spends less time planning compared to an inexperienced agent. Furthermore, in contrast to the previous accounts that propose ad-hoc [18] or optimal, but with very strong assumptions (i.e., MB tree-expansion has an infinite depth), [19] models for MB-MF arbitration mechanisms, our proposed model’s optimality is based on more reasonable assumptions.

Our algorithm further predicts that in a plan-until-habit scheme, time-limitation would reduce the depth of planning. That is, time pressure would monotonically limit the total number of branches to be expanded, pressing the agent to switch to habitual/heuristic values at a shallower depth. This is due to the fact that every tree-expansion step is assumed to take a certain amount of time, *ϵ*. Therefore, our model, for the first time, accounts for recent evidence showing that humans use a plan-until-habit scheme and that time pressure reduces their depth of MB planning [11], resulting to a relying on habitual responses at a shallower level.

In this experimental study [11], participants first learned the stationary transition structure of the environment in a three-step task. They then navigated through the decision tree, in each trial, to reach their desired terminal state. The rewarding value of the terminal states was non-stationary and changed along the trials, allowing to measure, from participants’ choices, whether or not they use a plan-to-habit scheme; and if they do, what depth of planning they adopt. The experiment imposed a decision time-limit of either 2000 or 700 milliseconds to two different groups of participants. While both groups showed a significant behavioral signature of plan-to-habit responding, participants that experienced a shorter time-limitation showed pruning the tree and switching to MF values at shallower levels.

### Plan-to-habit pruning in comparison

In this section, we qualitatively compare our plan-to-habit pruning algorithm to other methods, such as Monte Carlo tree search.

#### Mean-based pruning, variance-based pruning

Let us consider a simple pruning algorithm that expands the tree only according to the mean value of the strategies, and ignores their variances (e.g., the algorithm always—or stochastically- expands the strategy with the highest mean value, $\arg\max_{A}E\left[V\left(A\right)\right]$). The critical drawback of such algorithm is that it does not expand uncertain trajectories that have relatively smaller mean values. The true value of a strategy with a low estimated mean but high estimated uncertainty might be even higher than the strategy known to have the highest estimated mean. Therefore, uncertain strategies should be given the chance to prove their worth. In this sense, our algorithm proposes an optimal weighting of mean and variance in order to prioritize expansions.

Furthermore, note that an algorithm that only takes into account the mean values cannot explain the canonical experimental evidence of the gradual transition from goal-directed to habitual behavior over time [14–17]. Explaining such a transition, at least in all the existing accounts, requires keeping track of the MB and MF uncertainties, and taking them into account when arbitrating between the two systems [18, 19].

Similarly, an algorithm that expands the tree only on the basis of the uncertainty of trajectories’ values, would only favor mental exploration of uncertain trajectories, even when their low mean value renders them totally unpromising.

#### Monte Carlo tree search

Monte Carlo tree search (MCTS) is a family of algorithms that incrementally and stochastically builds a search tree to approximate state-action values. This incremental growth, as in our algorithm, prioritizes the promising regions of the search space by directing the growth of the tree towards high-value states.

A so-called tree policy is used to traverse the search tree and select a node which is not fully expanded, i.e., it has immediate successors that are not included in the tree. The node is then expanded by adding one of its unexplored children to the tree, from which a trajectory will be simulated for a fixed number of steps or until a terminal state is reached. Such trajectories are generated using a rollout policy which is typically fast to compute—for instance at each step of the trajectory actions are selected randomly and uniformly. The outcome of this trajectory (i.e., cumulative discounted rewards along the trajectory) is used to update the value estimates of the nodes in the tree that lie along the path from the root to the expanded node.

MCTS algorithms diverges from our approach mainly in how the value of states and actions are computed. The former relies on simulated experiences, called rollouts, whereas the latter relies on summaries of past experiences in terms of “cached” values (or model-free values). As such, the latter is much cheaper to compute, but is dependent on the policy with which those experiences are collected. In MCTS, however, values depend mostly on the tree policy, which is adaptive. Consequently, relying on past experiences, as in vur model, is cheaper but less flexible.

Our plan-to-habit pruning algorithm can be compared to MCTS methods on another level by focusing on tree policies. The most popular MCTS tree policy is “UCT” (Upper Confidence Bound 1 applied to trees) [20], which is based on a successful multi-armed bandit algorithm called “UCB1” (Upper Confidence Bound 1). UCB1 assigns scores to actions as a combination of their (empirical) mean returns and their exploration coefficients, which reflects how many times an action is sampled in comparison to other actions. UCT adapts this UCB1 rule to MCTS by recursively applying this rule to select actions down the tree starting from the root node.

UCT is simple and has successfully been utilized for many applications. However, it has also been noted [21, 22] that UCT’s goal is different from that of approximate planning. UCT attempts to ensure a high net *simulated* worth for the actions that are taken during the Monte Carlo simulations that comprise planning. However, all that actually matters is the *real* worth of the single action that is ultimately taken in the world after all the simulations have terminated. To put it in another way, in planning, simulations and expansions are valuable, only because they help select the best action. However, UCT actually aims to maximize the sum of rewards obtained in simulations, rather than paying direct attention to the quality of actual (i.e., not simulated) actions. Consequently, it tries to avoid simulations with potentially low rewards, even though they might help select better actions. In other words, even though UCT explicitly computes an “exploration bonus” that favors infrequently visited nodes, it still underestimates how valuable exploration is. In fact, it has been shown that modifying UCT to explore (asymptotically) more when selecting root actions increases its performance [21, 22]. Our model does not suffer from this problem of underexploration as it explictly quantifies the expected gain of expanding a node.

## Discussion

Finding optimal or near optimal actions requires comparing the expected value of all possible plans that can be taken in the future. This can be achieved by explicitly expanding a model that represents the underlying structure of the environment, followed by calculating the expected value of each plan. However, the computational complexity of this process grows exponentially with the depth of search for optimal plans, which makes it infeasible to implement in all but the smallest environments. Indeed, evidence shows that humans and other animals use alternative ways that have lower computational complexities than explicit search. Examples are using ‘cached’ values of actions instead of recalculating them at each decision point [18], or using ‘action chunking’, in which actions span over multiple future states [23]. Here, we suggest that such decision-making strategies are not operating independent of the planning processes, but they interact in order to provide a planning process that adapts its extent according to time and cognitive resource and therefore, scales to complex environments. In particular, the model that we suggest is built upon two bases: (i) the planning process is directed toward the parts of the environment’s model that are most likely to benefit from further deliberation, and (ii) the planning process uses ‘cached’ action values for the unexpanded (i.e., pruned) parts of the tree. Simulation results showed that the model prunes effectively in a synthetic grid world, and that it explains several patterns reported in humans/animals.

Namely, a sequential decision-making task has demonstrated that humans use strategies such as ‘fragmentation’ and ‘hoarding’, in addition to pruning, for efficient planning. The pruning process, however, was shown to play a significant role on the top of those strategies [13]. Indeed, the data shows that humans stop expanding a branch of the model once they encounter a large punishment. This effect was previously accounted for, in the model-based planning framework, by adding a new parameter that encodes the probability of stopping the search after encountering a large punishment. The model here does not explicitly contain such a parameter, but the pruning effect emerges naturally based on the fact that the value of uncertainty resolution is lower for the branches of the model that start with large punishments and therefore, they are more likely to be pruned.

Another component of the model here is using the cached values for unexpanded parts of the model, which is in line with previous works [11, 12]. The psychological nature of such cached values can be related to either Pavlovian (as used in [12]) or instrumental (as used in [11]) processes in the brain, depending on whether cached values are coded for state or for state-action pairs, respectively. In the former case, our algorithm represents a collaborative interaction between instrumental model-based and Pavlovian processes [24]. In the latter case, it represents interaction between instrumental model-based and instrumental model-free processes. The theoretical framework we presented here is readily compatible with either case.

As discussed in the previous sections, temporal discounting of future rewards (and punishments) is a necessary component in the current framework. Reduction of uncertainty is a variable that changes monotonically with the discount factor: the smaller the *γ*, the less dependence of the value of each strategy on uncertain cached values on the leaves and therefore, the more reduction of uncertainty by deepening the tree. However, when a new piece of information on a leaf at depth *d* is achieved, its policy-improvement impact on the root-level actions is measured at the root of the tree, thus discounted by a factor *γ*^*d*^. Therefore, the smaller the *γ* is, the less valuable a given uncertainty reduction is. This effect counteracts the above-mentioned effect of *γ* on the degree of uncertainty reduction. As a result, discount factor has a non-monotonic effect on vur and thus, on the depth of planning. vur is equal to zero for *γ*-values of zero and one, and reaches a maximum for an intermediate value of *γ* (its exact value depends on other parameters).

In sum, we proposed a principled algorithm for pruning in a plan-until-heuristic scheme. While we showed the ability of the model in accounting for several behavioral patterns in humans/animals, whether or not people use such algorithm requires further direct experiments. Such experiments could test the effect of variables like the mean and the variance of cached values on the probability of expanding a node. On the theoretical front, our algorithm can benefit from several improvements, most notably, from relaxing the assumption that the environment has a deterministic transition structure. In that case, the algorithm could increase the efficiency of the state-of-the-art algorithms that use a plan-until-heuristic scheme in complex games [10]. Furthermore, whereas we simply assume here that planning and action execution cannot be performed in parallel, it is reasonable to assume that agents deliberate over upcoming choices while performing previously chosen actions.

## Methods

We focus on deterministic Markov decision processes (MDPs). The environment is composed of a finite set of states S; a finite set of actions A; a (potentially partial) transition function T:S×A⇸S; and a reward function $f_{R}:S\times A\times S\to R$. The agent interacts with the environment via a (potentially stochastic) policy π:S×A⇸[0,1] s.t. ∑_*a*_ *π*(*s*, *a*) = 1 for all *s*, with the goal of maximizing the expected value of the cumulative discounted rewards $E[R_{t}|s_{t}=s]$, where $R_{t}=\sum_{i=0}^{\infty }\gamma^{i}r_{t+i}$, *s* is the start state, and *γ* is the discount factor. The state-action values of a policy *π* are defined as $Q^{\pi }\left(s,a\right)=E_{\pi }[R_{t}|s_{t}=s,a_{t}=a]$. Finally, the optimal state-action values are defined as Q*(*s*, *a*) = max_*π*_ *Q*^*π*^(*s*, *a*).

We assume for now that the model-based (MB) system has perfect knowledge of the environment (i.e., the reward and transition functions) (we will relax this assumption later). The agent uses some of this information to build a search tree representation, which relates the current state *s*_*t*_ to other states that can potentially be occupied in the future. The root of the tree is *s*_*t*_, and its immediate children include the one-step-reachable states.

Let us illustrate the formation of a search tree. The agent creates a tree node, containing information about her current state *s*_*t*_, which becomes the root of the tree, meaning all other nodes will stem directly or indirectly from it. The agent picks an action *a* available at *s*_*t*_ to expand, which in turn adds *s*′ ≔ *T*(*s*_*t*_, *a*) to the tree as a child node of *s*_*t*_. Now, if the agent continues planning, she can either expand an action from *s*_*t*_, assuming there are more than one action available at *s*_*t*_, or she can choose to expand from *s*′. The planning process is composed of iteratively selecting an action to expand from the set of unexplored node-action pairs and adding the resulting new state to the tree as a new node.

Let us consider the state of a tree at a given time, containing a total number of *n* unexpanded node-action pairs. This means, there are *n* trajectories that start from *s*_*t*_ and terminate at one of the unexpanded state-action pairs. We call each trajectory a “strategy”, denoted by *A*_*i*_, which is a tuple of state-action pairs, and introduce the search frontier *F* = {*A*_1_, *A*_2_, …, *A*_*n*_} as the set of all strategies for a given tree. We define *expanding* a strategy *A* by adding *s*′, the immediate successor state of the unexplored state-action pair at the end of *A*, to the tree and adding the resulting new strategies to the frontier. These new strategies have the form *A* + 〈*s*′, *a*′〉, where *a*′ denotes any action available at *s*′, and + is a tuple-concatenation operator. Note that after the expansion, if *A* is no longer unexplored—that is, has no unexpanded actions—then *A* is removed from *F*. This process of tree expansion goes on until an action is taken or the frontier is empty. The latter condition means the tree captures all possible trajectories in the MDP, which can only happen in an episodic MDP where no matter what actions the agent takes, she ends up in a terminal state (i.e., the state that ends the episode) after a finite number of actions.

We also assume that the agent has an estimation of the expected cumulative discounted rewards of each state-action pair 〈*s*, *a*〉, encoded by a random variable *Q*(*s*, *a*). A model-free (MF) system, for example, can represent such *Q*-values as random normal variables by tracking the first order statistics (i.e., mean) and second order statistics (i.e., variance) of the values [25, 26]. Given that state-action values are the *expected* longterm discounted rewards, any stochastic estimation of it will be normally distributed given the Central Limit Theorem assuming a fixed sampling policy and a reasonable (*f*_*R*_ has finite variance for all 〈*s*, *a*, *s*′〉) reward structure. Thus, it is reasonable to represent *Q*’s as random normal variables. With these settings, and in keeping with the plan-until-habit scheme, the value of a strategy *A*_*i*_ that ends with an 〈*s*_*M*_, *a*_*M*_〉 at depth *M* with $Q\left(s_{M},a_{M}\right)∼N\left(\mu_{s_{M},a_{M}},\sigma_{s_{M},a_{M}}^{2}\right)$ can be estimated by

$$\begin{array}{c} V\left(A_{i}\right)=r_{1}+\gamma r_{2}+\gamma^{2}r_{3}+\cdots +\gamma^{M-1}r_{M}+\gamma^{M}Q\left(s_{M},a_{M}\right)\;, \end{array}$$ (3)

where each *r*_*i*_ corresponds to the MB estimation of reward after taking the *i*^*th*^ action in the strategy. Assuming that there is no uncertainty in estimating the immediate rewards (As discussed later, it is straightforward to relax the assumption of zero uncertainty for immediate rewards), *r*_1_, *r*_2_, .., *r*_*M*_, the total variance of $V\left(A_{i}\right)∼N\left(\mu_{i},\sigma_{i}^{2}\right)$ is $\sigma_{i}^{2}=\gamma^{2M}\sigma_{s_{M},a_{M}}^{2}$. It can be seen that as a strategy gets deeper, MF value distributions (i.e., *Q*’s) get discounted more, which will form the basis of our method.

We seek to compute the value of expanding the tree along *A*_*i*_. The agent knows that expanding *A*_*i*_ will lead to a new, yet unknown state, *s*_*M*+1_, where an action *a*_*M*+1_ with the highest *Q*-value, *Q*(*s*_*M*+1_, *a*_*M*+1_), among other actions of that state exists. This potential expansion will lead to a new strategy, $A_{i}^{*}$, with its value estimated by:

$$\begin{array}{c} V\left(A_{i}^{*}\right)=r_{1}+\gamma r_{2}+\gamma^{2}r_{3}+\cdots +\gamma^{M-1}r_{M}+\gamma^{M}r_{M+1}+\gamma^{M+1}Q\left(s_{M+1},a_{M+1}\right)\;. \end{array}$$ (4)

Note that *r*_*M*+1_, *s*_*M*+1_, *a*_*M*+1_, and *Q*(*s*_*M*+1_, *a*_*M*+1_) are unknown prior to expansion. To reflect this, we use the notation $\bar{V}\left(.\right)$ to denote an unknown value estimation:

$$\begin{array}{c} \bar{V}\left(A_{i}^{*}\right)=r_{1}+\gamma r_{2}+\gamma^{2}r_{3}+\cdots +\gamma^{M-1}r_{M}+\gamma^{M}{\bar{r}}_{M+1}+\gamma^{M+1}\bar{Q}\left({\bar{s}}_{M+1},{\bar{a}}_{M+1}\right)\;, \end{array}$$ (5)

where ${\bar{r}}_{M+1}$ and $\bar{Q}\left({\bar{s}}_{M+1},{\bar{a}}_{M+1}\right)$ denote, respectively, the immediate reward and the value distribution of the successor state-action pair, both unknown prior to expansion and thus, denoted with a bar (¯). Intuitively, $E[V\left(A_{i}\right)]$ should be equal to $E[\bar{V}\left(A_{i}^{*}\right)]$, because they result from the same information prior to an expansion. Only with the extra information obtained from an expansion, namely after observing ${\bar{r}}_{M+1}$ and $\bar{Q}\left({\bar{s}}_{M+1},{\bar{a}}_{M+1}\right)$, the agent hopes to gain precision. In fact, we assume the agent’s probability estimates are coherent in the sense that her expectations of ${\bar{r}}_{M+1}$ and $\bar{Q}\left({\bar{s}}_{M+1},{\bar{a}}_{M+1}\right)$ are in line with $E[V\left(A_{i}\right)]$. Therefore, we have:

$$\begin{array}{c} E\left[V\left(A_{i}\right)\right]=E_{\bar{Q},\bar{r}}[E[\bar{V}\left(A_{i}^{*}\right)|\bar{Q},\bar{r}]]\;\;, \end{array}$$ (6)

where we drop the subscript *M* + 1 of *r* and arguments ${\bar{s}}_{M+1},{\bar{a}}_{M+1}$ of $\bar{Q}$ for brevity. This equality is also known as the law of total expectation, and here it suggests that an expansion may change the expected value of $V(A_{i}^{*})$ but not *in expectation*. We should emphasize that an agent does not necessarily need to obey this, but not doing so might result in inefficiencies. Particularly, if Eq 6 is not obeyed, then a Dutch book may be formed such that the agent would expect to lose value by performing tree expansions.

Also, note that,

$$\begin{array}{c} {\text{Var}}_{\bar{Q},\bar{r}}[E[\bar{V}\left(A_{i}^{*}\right)|\bar{Q},\bar{r}]]\geq \text{Var}[E[V\left(A_{i}\right)]]=0\;, \end{array}$$ (7)

which means that while the agent knows the exact mean of *A*_*i*_’s value ($\text{Var}[E[V\left(A_{i}\right)]]=0$), the mean of the new strategy’s value is unknown prior to expansion. This variability in the expected value of the new strategy creates the possibility that the true (i.e., after expansion) expected value of $A_{i}^{*}$ is even higher than the mean value of the best currently-expanded strategy. In fact, prior to expansion, the agent believes that acting on the basis of its currently-expanded tree will pay her $\max_{A\in F}E\left[V\left(A\right)\right]$, which is the mean value of the best strategy. However, if the true expected value of $A_{i}^{*}$ is even higher than $\max_{A\in F}E\left[V\left(A\right)\right]$, then the agent can change her policy and “gain” extra reward. The expectation of this “gain”, given the distribution over the expected value of $A_{i}^{*}$, computes the value of expanding a strategy. In other words, expanding a strategy will yield a net expected increase (assuming the expanded strategy has variance in its value) in the expected value of the best strategy, which we refer to as the *value of uncertainty resolution* (vur). The vur along the strategy *A*_*i*_ is equal to the expected value of policy improvement-induced reward resulting from observing ${\bar{r}}_{M+1}$ and $\bar{Q}\left({\bar{s}}_{M+1},{\bar{a}}_{M+1}\right)$ Formally, given the current state of the search frontier *F*, vur(*A_i_*|*F*) is simply the difference between the expected value of best strategy *after* expanding *A*_*i*_ (i.e., observing ${\bar{r}}_{M+1}$ and $\bar{Q}\left({\bar{s}}_{M+1},{\bar{a}}_{M+1}\right)$) and *before* expanding *A*_*i*_:

$$\text{VUR}(A_{i}|F)=E_{\bar{Q},\bar{r},}[\max(E[\bar{V}\left(A_{i}\right)|\bar{Q},\bar{r}],\max_{A\in F-A_{i}}E[V(A)])]-\max_{A\in F}E\left[V\left(A\right)\right]$$ (8)

$$\begin{array}{cc} & \geq 0\;. \end{array}$$ (9)

where *F* − *A*_*i*_ is the set *F* excluding *A*_*i*_ assuming *A*_*i*_ will be fully explored after expansion, and thus be removed from *F*. Otherwise, the max should run over *F*. The second (with minus) term in Eq 8 is the expected value of the best strategy in the frontier. The first term is the expected value of the best strategy after expansion. The vur is always non-negative because of Jensen’s inequality: max is convex and thus, the expectation of the max of random variables has to be larger than or equal to the maximum of expectations.

In order to progress further analytically, we make an assumption and assert that Var[*Q*(*s*_*M*_, *a*_*M*_)] = Var[*Q*(*s*_*M*+1_, *a*_*M*+1_)]. That is, we assume that MF value distributions for 〈*s*, *a*〉 and its immediate successor state-action pairs have the same uncertainty, possibly because the habitual system has had a similar number of experiences (i.e., samples) of neighboring actions and they are possibly of similar values. We can see in Eq 4 that only *Q*(*s*_*M*+1_, *a*_*M*+1_) contributes to the uncertainty in $V(A_{i}^{*})$. Therefore we have,

$$V(A_{i}^{*})∼N(\mu_{i}^{*},\gamma^{2M+2}\sigma_{s_{M+1},a_{M+1}}^{2})$$ (10)

$$\begin{array}{cc} & =N(\mu_{i}^{*},\gamma^{2}(\gamma^{2M}\sigma_{s_{M},a_{M}}^{2})) \end{array}$$ (11)

$$\begin{array}{cc} & =N(\mu_{i}^{*},\gamma^{2}\sigma_{i}^{2})\;, \end{array}$$ (12)

where $\mu_{i}^{*}\in R$ is the mean, which we will obtain shortly, and $\sigma_{i}^{2}=\gamma^{2M}\sigma_{s_{M},a_{M}}^{2}$ is the variance of *V*(*A*_*i*_). However, both *V*(*A*_*i*_) (magenta curve in Fig 1C) and $V(A_{i}^{*})$ (black/grey curves in Fig 1C) are estimating the value for the same action at the root state, *s*_*t*_. Therefore, the value distributions $V\left(A_{i}\right)∼N\left(\mu_{i},\sigma_{i}^{2}\right)$ and $V\left(A_{i}^{*}\right)∼N(\mu_{i}^{*},\gamma^{2}\sigma_{i}^{2})$ should be consistent as in Eq 6, implying

$$\begin{array}{c} V\left(A_{i}\right)=E_{\mu_{i}^{*}}\left[V\left(A_{i}^{*}\right)\right]\;, \end{array}$$ (13)

which can only be satisfied if

$$\begin{array}{c} \mu_{i}^{*}∼N\left(\mu_{i},\left(1-\gamma^{2}\right)\sigma_{i}^{2}\right)\;. \end{array}$$ (14)

The distribution over $\mu_{i}^{*}$ represents the probability distribution of the expected value of a strategy after expansion. This variability comes from the fact that we will have additional pieces of information, namely *r*_*M*+1_ and *Q*(*s*_*M*+1_, *a*_*M*+1_).

Note that in equation Eq 10, the only source of variance in $A_{i}^{*}$ is assumed to be the variance in *Q*(*s*_*M*+1_, *a*_*M*+1_). In other words, the agent is assumed to have no uncertainty in estimating *r*_1_, *r*_2_, .., and *r*_*M*_. It is straightforward to relax this assumption by keeping track of the variance of *r*_1_, *r*_2_, .., and *r*_*M*_, denoted by $\sigma_{r_{1}}^{2},\sigma_{r_{2}}^{2},..,\sigma_{r_{M}}^{2}$. In that case, Eq 10 will be replaced by

$$V(A_{i}^{*})∼N(\mu_{i}^{*},\sigma_{r_{1}}^{2}+\gamma^{2}\sigma_{r_{2}}^{2}+..+\gamma^{2M}\sigma_{r_{M+1}}^{2}+\gamma^{2M+2}\sigma_{s_{M+1},a_{M+1}}^{2})$$ (15)

$$\begin{array}{cc} & =N(\mu_{i}^{*},\sigma_{i}^{2}-\gamma^{2M}\left(\left(\gamma^{2}-1\right)\sigma_{s_{M},a_{M}}^{2}+\sigma_{r_{M}}^{2}\right))\;, \end{array}$$ (16)

which gives

$$\begin{array}{c} \mu_{i}^{*}∼N(\mu_{i},\gamma^{2M}\left(\left(1-\gamma^{2}\right)\sigma_{s_{M},a_{M}}^{2}-\sigma_{r_{M}}^{2}\right)))\;, \end{array}$$ (17)

where $\sigma_{s_{M},a_{M}}^{2}=\gamma^{-2M}\sigma_{i}^{2}$ again.

This will take MB imperfection information about the reward function into account. Eq 10 also assumes that the agent has perfect information regarding the transition function. Given that our algorithm is only developed for MDPs with deterministic transition function, this assumption is feasible. Relaxing these assumptions (i.e., deterministic, and perfect knowledge of, transition function) are left for future work.

Relaxing the assumption on deterministic transition function would result the estimated value, $V(A_{i}^{*})$, of the strategy $A_{i}^{*}$ to become a mixture of Gaussians, rather than a simple Gaussian distribution. Computing $\mu_{i}^{*}$ and vur for such cases would significantly increase the computational cost of meta-cognition and hence, developing approximation methods would be required. For example, one could resort to Monte Carlo methods, where a set of transitions are sampled from the stochastic transition function, over which the vur is averaged.

Given we now know the distribution of $\mu_{i}^{*}$, we can rewrite the vur definition given in Eq 8:

$$\begin{array}{c} \text{VUR}\left(A_{i}|F\right)=E_{\mu_{i}^{*}}[\max(\mu_{i}^{*},\max_{A\in F-A_{i}}E[V(A)])]-\max_{A\in F}E[V(A)]\;, \end{array}$$ (18)

where $\mu_{i}^{*}$ is distributed according to Eq 14 and *F* − *A*_*i*_ is the set *F* excluding *A*_*i*_. We show in S1 Text that there is a closed-form solution for vur(*A_i_*|*F*) defined above.

Utilizing this uncertainty resolution mechanism, the agent can simply find the most promising strategy to expand, via $\arg\max_{A_{i}\in F}\text{VUR}\left(A_{i}|F\right)$. The agent can continue expanding the search tree by reducing the uncertainties of the most promising branches until the value gained by expansion is less than the opportunity cost of expanding (as in [19]), or the search can continue until the working memory is full. The latter termination condition could be implemented based on the assumption that the working memory has a limited number of slots [27, 28] (e.g., for storing states of the expanded tree). Alternatively, one could assume that the working memory is inherently corrupted by noise, and that the level of this noise increases with the number of items in memory [29]. It is straightforward to incorporate this mechanism into our algorithm: expansion results in the variance of $V(A_{i}^{*})$ to decrease by a factor *γ*^2^, but also increases by an additive factor that is proportional to the number of items (e.g. states) currently stored in the working memory. Thus, one can compute when the noise overwhelms the resolved uncertainty.

It is noteworthy that in this paper, computing vur is based on the assumption that when the value of expansion is bigger than its cost and thus an expansion should occur, an action will be executed immediately after that expansion. In fact, our model does not compute the value of further expansions following the next potential expansion. Relaxing this assumption would require computing the value of expanding all *subsets* of available and potentially-emerging strategies. In this case, for a certain subset like *T*_1_, *T*_2_, one needs to compute vur(*T*_1_, *T*_2_|*F*) and compare it with *B*.*C*, where *B* = 2 is the number of expansions being considered, and *C* is the cost of one single expansion. We show in S1 Text (section “on considering vur values independently”) that the value of expanding several strategies before performing an action is not necessarily equal to the sum of the value of expanding each of those strategies independently. In general, computing the optimal sequence of expansions for a budget of B would be NP-complete in B, as it reduces to stochastic knapsack problem [30].

Another interesting outcome of this model is that the relationship between vur and *γ* roughly follows an inverse U-shaped curve. If *γ* = 0, then *V*(*A*) as given in Eq 3 will be a scalar; as such, vur will be 0. If *γ* = 1, then the variance of $E[V\left(A^{*}\right)]$ as given Eq 14 will be zero, which too will result in vur being 0. The interpretation of these conditions is easy: if you do not care about the future, then no need to plan; and in the latter condition, the agent cannot gain precision by discounting the model-free estimates.

## Supporting information

S1 Text Proofs and derivations. We provide vur-related proofs and derivations.
(PDF)
