# Supplementary material for: Optimizing the depth and the direction of prospective planning using information values
Source: PLoS Comput Biol. 2019 Mar 12;15(3):e1006827. doi: 10.1371/journal.pcbi.1006827 (PMC6440644; doi:10.1371/journal.pcbi.1006827)
Supplement: S1 Text — We provide vur-related proofs and derivations. (PDF) [file pcbi.1006827.s001.pdf]

Supporting Information

Closed form of VUR

In the case of normally distributed value functions, it is possible to further expand the VUR as defined in Eq M.18[1]. Let us denote the indices of the best and the second-best actions by α and β. respectively. That is, $A_\alpha = \arg\max_{A_i \in F} \mathbb{E}\left[V(A_i)\right]$ and $A_\beta = \arg\max_{A_i \in F - A_\alpha} \mathbb{E}\left[V(A_i')\right]$. Then we can transform Eq M.18 into

$$\text{VUR}(A_i|F) = \begin{cases} \mathbb{E}_{\mu_i^*}\left[\max\left(\mu_i^*, \mu_\beta\right)\right] - \mu_\alpha & \text{if } i = \alpha \\ \mathbb{E}_{\mu_i^*}\left[\max\left(\mu_i^*, \mu_\alpha\right)\right] - \mu_\alpha & \text{otherwise} \end{cases} . \quad (1)$$

We also have

$$\begin{aligned} \mathbb{E}_{\mu_i^*}\left[\max\left(\mu_i^*, \mu_\beta\right)\right] &= P(\mu_i^* > \mu_\beta)\mathbb{E}[\mu_i^*|\mu_i^* > \mu_\beta] + P(\mu_i^* \leq \mu_\alpha)\mu_\beta & (2) \\ &= P(\mu_i^* > \mu_\beta)\mathbb{E}[\mu_i^*|\mu_i^* > \mu_\beta] + (1 - P(\mu_i^* > \mu_\alpha))\mu_\beta & (3) \\ &= P(\mu_i^* > \mu_\beta)\left(\mathbb{E}[\mu_i^*|\mu_i^* > \mu_\beta] - \mu_\beta\right) + \mu_\beta \, . & (4) \end{aligned}$$

Given $\mu_i^* \sim \mathcal{N}(\mu_i, (1-\gamma^2)\sigma_{A_i}^2)$, we can use the properties of truncated normal distributions to get a closed form solution. Let us first define $\zeta_i^\beta = \frac{\mu_i - \mu_\beta}{\sigma_i}$ and $\zeta_i^\alpha = \frac{\mu_i - \mu_\alpha}{\sigma_i}$, where σ_i is the standard deviation of μ_i^*, that is $\sigma_i = \sqrt{(1-\gamma^2)\sigma_{A_i}^2}$. Then we get,

$$\begin{aligned} \mathbb{E}_{\mu_i^*}\left[\max\left(\mu_i^*, \mu_\beta\right)\right] &= \Phi(-\zeta_i^\beta)\left(\mu_i + \sigma_i \frac{\phi(\zeta_i^\beta)}{1 - \Phi(\zeta_i^\beta)} - \mu_\beta\right) + \mu_\beta & (5) \\ &= \Phi(-\zeta_i^\beta)(\mu_i - \mu_\beta) + \sigma_i \phi(\zeta_i^\beta) + \mu_\beta & (6) \\ &= \sigma_i \left[\phi(\zeta_i^\beta) - \zeta_i^\beta \Phi(-\zeta_i^\beta)\right] + \mu_\beta \, , & (7) \end{aligned}$$

$$\mathbb{E}_{\mu_i^*}\left[\max\left(\mu_i^*, \mu_\alpha\right)\right] = \sigma_i\left[\phi(\zeta_i^\alpha) - \zeta_i^\alpha \Phi(-\zeta_i^\alpha)\right] + \mu_\alpha \, . \quad (8)$$

Finally, combining these, we obtain

$$\text{VUR}(A_i|F) = \begin{cases} \sigma_i[\phi(\zeta_i^\beta) - \zeta_i^\beta \Phi(-\zeta_i^\beta)] + \mu_\beta - \mu_\alpha & \text{if } i = \alpha \\ \sigma_i[\phi(\zeta_i^\alpha) - \zeta_i^\alpha \Phi(-\zeta_i^\alpha)] & \text{else} \end{cases} . \quad (9)$$

Higher variance means higher VUR

Here, we show that $\text{VUR}(A_i|F)$ decreases monotonically as σ_i decreases. Let us focus on the second condition of VUR as defined in Eq M.9. Let

[1] Prefix M. refers to the equation in the main text.

$$f(\sigma_i) = \sigma_i[\phi(\zeta_i^\alpha) - \zeta_i^\alpha \Phi(-\zeta_i^\alpha)] \,. \tag{10}$$

and $x = \zeta_i^\alpha \sigma_i$. Then,

$$f(\sigma_i) = \sigma_i[\phi(x/\sigma_i) - x/\sigma_i \Phi(-x/\sigma_i)] \tag{11}$$

$$\frac{df}{d\sigma_i} = \phi(x/\sigma_i) - x/\sigma_i \phi'(x/\sigma_i) + (x/\sigma_i)^2 \phi(x/\sigma_i) \tag{12}$$

$$= \phi(x/\sigma_i) + (x/\sigma_i)^2 \phi(x/\sigma_i) + (x/\sigma_i)^2 \phi(x/\sigma_i) \tag{13}$$

$$= \phi(x/\sigma_i) \left(2(x/\sigma_i)^2 + 1\right) \tag{14}$$

$$\geq 0 \,. \tag{15}$$

The same also holds for the first condition of VUR, which we omit here.

Asymptotic complexity of VUR-greedy

Here, we show that computing the VUR-greedy policy requires a constant number of operations asymptotically in expectation, under some mild conditions. That is, as the search tree grows larger, the expected cost of finding the best strategy remains constant asymptotically.

Assume the search tree has a constant branching factor b such that $b > 1$, and has a height (or depth) of h. Also assume that all the leaves are at the same level—i.e., there are b^h leaves in total. Note that, while computing the VUR for all of the leaves, we need μ_α for $b^h - 1$ nodes, and μ_β for 1 node. That is, if μ_α changes after an expansion, VUR values for all the leaves need to be recomputed. If not, then only a single (or two) recomputations are sufficient. One for the expanded strategy, and one for μ_β if the expanded strategy turns out to have the second highest mean.

Assume A is the strategy we consider expanding, which would result in A^*. Then we can calculate the expected number of recomputations as a function of depth after expanding A as,

$$C(h) = P(\mathbb{E}[A^*] > \mu_\alpha) b^h + \text{const} \tag{16}$$

where the const term in the above equation contains the terms that do not depend on h. Let x be the distance between μ_A and μ_α, i.e. $x = \mu_\alpha - \mu_A$. Then, we have,

$$C(h) = \Phi(-x/\sigma_A) b^h + \text{const} \,. \tag{17}$$

σ_A will decrease as h increases because of the discounting factor γ^h. Assume $\sigma_A = \gamma^h \sigma$. Then, we have,

$$C(h) = \Phi(-x\gamma^{-h}\sigma^{-1}) b^h + \text{const} \,. \tag{18}$$

Note that $\lim_{h\to\infty} \Phi(-x\gamma^{-h}\sigma^{-1}) = 0$ whereas $\lim_{h\to\infty} b^h = \infty$. Using L'Hôpital's

rule we have,

$$\lim_{h\to\infty} \frac{\Phi(-x\gamma^{-h}\sigma^{-1})}{b^{-h}} = \lim_{h\to\infty} \frac{d\Phi(-x\gamma^{-h}\sigma^{-1})/dh}{db^{-h}/dh} \tag{19}$$

$$= \lim_{h\to\infty} \frac{\phi(-x\gamma^{-h}\sigma^{-1})(x\sigma^{-1}\gamma^{-h}\ln\gamma)}{-b^{-h}\ln b}. \tag{20}$$

Note that this term is positive as both the numerator and the denominator are negative. Thus, we can take the log of the term inside the limit,

$$\ln\left(\frac{\phi(-x\gamma^{-h}\sigma^{-1})(x\sigma^{-1}\gamma^{-h}\ln\gamma)}{-b^{-h}\ln b}\right) = -x^2\sigma^{-2}\gamma^{-2h}/2 - h\ln(\gamma) - h\ln(b) + \text{const}. \tag{21}$$

We can see that,

$$\lim_{h\to\infty} \ln\left(\frac{\phi(-x\gamma^{-h}\sigma^{-1})(x\sigma^{-1}\gamma^{-h}\ln\gamma)}{-b^{-h}\ln b}\right) = -\infty\,. \tag{22}$$

Given we can exchange the limit with the logarithm, we see

$$\lim_{h\to\infty} \frac{\Phi(-x\gamma^{-h}\sigma^{-1})}{b^{-h}} = 0\,. \tag{23}$$

Therefore, we only need a constant number of operations on average to evaluate $\arg\max_A \text{VUR}(A|F)$ in the limit of the search tree depth.

On considering VUR values independently

As mentioned in the main text, one possible limitation of our pruning proposal is that it might be a better idea to search over the combinations of possible expansions, rather than considering expansions in isolation. That is, to expand

$$\arg\max_{A\subseteq F} \text{VUR}(A|F)\,, \tag{24}$$

instead of

$$\arg\max_{A_i\in F} \text{VUR}(A_i|F)\,. \tag{25}$$

Here, we give a partial argument why this likely is not the case. If it can be shown that resolving uncertainties of two (or more) strategies *at the same time* is more valuable than the value obtained by considering those strategies *separately* and *sequentially*, this would imply action selection via Eq M.25 leaves some information value on the table.

Let us consider a simple case, where we consider expanding two strategies at once, which yields

$$\text{VUR}(A_i, A_j|F) = \mathbb{E}_{\mu_i^*,\mu_j^*}\left[\max(\mu_i^*, \mu_j^*, \mu_\alpha)\right] - \mu_\alpha\,, \tag{26}$$

where we assume $i \neq \alpha$ and $j \neq \alpha$ to avoid complications caused by the piecewise nature of VUR. However, the argument holds for the general case as well.

Is it possible that $\text{VUR}(A_i, A_j|F) > \text{VUR}(A_i|F) + \mathbb{E}[\text{VUR}(A_j|F, A_i)]$, where $\mathbb{E}[\text{VUR}(A_j|F, A_i)]$ is the expected information value of expanding A_j after expanding A_i? The answer is no, as we will show in the following argument.

Let us now define the conditional VUR, which in this case assumes A_i is expanded and a μ_i^* that is a more accurate estimate of $\mathbb{E}[V(A_i)]$ than μ_i is obtained,

$$\text{VUR}(A_j|F, A_i) = \mathbb{E}_{\mu_j^*}\left[\max(\mu_i^*, \mu_j^*, \mu_\alpha)\right] - \max(\mu_i^*, \mu_\alpha)\,. \tag{27}$$

However, since we would like to assess this conditional value prior to expanding A_i, the measure of our concern is,

$$\mathbb{E}\left[\text{VUR}(A_j|F, A_i))\right] = \mathbb{E}_{\mu_i^*}\left[\mathbb{E}_{\mu_j^*}\left[\max(\mu_i^*, \mu_j^*, \mu_\alpha)\right] - \max(\mu_i^*, \mu_\alpha)\right]\,. \tag{28}$$

Therefore,

$$\begin{aligned}
\text{VUR}(A_i|F) + \mathbb{E}\left[\text{VUR}(A_j|F, A_i))\right] &= \mathbb{E}_{\mu_i^*}\left[\max\left(\mu_i^*, \mu_\alpha\right)\right] - \mu_\alpha & (29)\\
&\quad + \mathbb{E}_{\mu_i^*}\left[\mathbb{E}_{\mu_j^*}\left[\max(\mu_i^*, \mu_j^*, \mu_\alpha)\right] - \max(\mu_i^*, \mu_\alpha)\right] & (30)\\
&= \mathbb{E}_{\mu_i^*}\left[\mathbb{E}_{\mu_j^*}\left[\max(\mu_i^*, \mu_j^*, \mu_\alpha)\right]\right] - \mu_\alpha & (31)\\
&= \text{VUR}(A_i, A_j|F)\,. & (32)
\end{aligned}$$

This can be seen as a chain rule for VUR and implies that there isn't anything to gain from expanding two strategies at once rather then expanding them sequentially. In fact, simultaneous expansion is in practice worse, since sequential expansion enables changing ones mind about what the best strategy to expand is after the expansion of another strategy.

Conjugate normal prior

In both of our experiments, we use conjugate normal priors to estimate Q-value distributions of the agent. We assume the *true* state-action values, $Q^\ddagger$, are distributed according to a known normal distribution. Formally, $Q^\ddagger(s,a) \sim \mathcal{N}(\mu_0, \sigma_0^2)$ i.i.d. for all $\langle s, a\rangle$ with known prior hyperparameters μ_0 and σ_0. The agent estimates would like to estimate $Q^\ddagger$, but she can only observe imperfect samples of it, in terms of cumulative trajectory returns in our case, $R_{sa} \sim \mathcal{N}\left(Q^\ddagger(s,a), \sigma^2\right)$ i.i.d., where R_{sa} is the cumulative discounted reward of a trajectory. Instead of assuming a fixed σ, we use the empirical standard deviation of the returns (i.e., of R_{sa}'s). One can easily show that this is equivalent to using a Normal-Gamma prior (as in [1]) and choosing the σ with the highest likelihood in the limit of the sample size. Then the $Q(s,a)$ used in our formulation is essentially the posterior $Q^\ddagger(s,a)$ condition on a sequence of trajectory rewards.

References

[1] Dearden R, Friedman N, Russell S. Bayesian Q-learning. In: Proceedings of the Fifteenth National/Tenth Conference on Artificial Intelligence/Innovative Applications of Artificial Intelligence. AAAI '98/IAAI '98. Menlo Park, CA, USA: American Association for Artificial Intelligence; 1998. p. 761–768.
